# A Circular Formation Method for Biomimetic Robotic Fish Inspired by Fish Milling

**DOI:** 10.3390/biomimetics8080583

**Published:** 2023-12-01

**Authors:** Ziye Zhou, Jincun Liu, Shihan Kong, Junzhi Yu

**Affiliations:** 1 China Academy of Aerospace Science and Innovation, Beijing 102600, China; 2 State Key Laboratory for Turbulence and Complex System, Department of Advanced Manufacturing and Robotics, College of Engineering, Peking University, Beijing 100871, China; kongshihan@pku.edu.cn; 3 College of Information and Electrical Engineering, China Agricultural University, Beijing 100083, China; 4 Science and Technology on Integrated Information System Laboratory, Institute of Software, Chinese Academy of Sciences, Beijing 100190, China

**Keywords:** circular motion, fish milling, multi-robot formation, biomimetic robotic fish

## Abstract

Circular motion phenomena, akin to fish milling, are prevalent within the animal kingdom. This paper delineates two fundamental mechanisms underlying such occurrences: forward following and circular topological communication. Leveraging these pivotal concepts, we present a multi-agent formation circular model based on a second-order integrator. This model engenders the attainment of homogeneous intelligence convergence along the circumferential trajectory. The convergence characteristics are intricately linked to the number of agents and the model parameters. Consequently, we propose positive and negative solutions for ascertaining the convergent circle property and model parameters. Furthermore, by integrating our proposed formation control methodology with a robotic fish dynamics model, we have successfully implemented simulations and experiments, demonstrating the circular formation of multiple biomimetic robotic fish. This study provides a mathematical explication for the circular motion observed in animal groups and introduces a novel approach to achieving circular formation in multiple robots inspired by biological phenomena.

## 1. Introduction

An intertwined and causal progression relationship exists between biological collectives, collective models, and multi-robot formation. Researchers draw inspiration from biological collective phenomena, establish corresponding group models, and apply them to multi-robot formation tasks. Among these, the step from natural collective phenomena to artificial group models is the most crucial. During this process, group models based on simple rules serve as a bridge to explore the mechanisms of animal collective motion. It is generally believed that the closer the simulation results of group models align with the observations of reality, the closer these simple rules that constitute the models come to the essence of biological collective motion.

Since Rynalods introduced the Boids rules (cohesion, separation, and alignment) in 1987, many studies in the field of animal collective behavior have considered “alignment”, which refers to the consistency of movement direction, as a critical factor in the formation of group behavior [1]. As a result, numerous related models have emerged [2,3,4,5]. Among them, the most influential is the group model proposed by Vicsek in 1995, whose model considers self-propelled particles moving on a plane and coordinates neighbors’ interactions within a certain distance based on the alignment rule to achieve overall consistency motion [2].

In the research field of control theory, starting from the Vicsek model, Morse et al. used non-negative matrices and stability theory to discuss the consensus problem of discrete systems with time-varying topology in 2003 [6]. Subsequently, in 2004, Murray et al. researched the consensus problem of switching topology and time-delay systems [7]. The studies by Morse and Murray ignited a research trend in the field of control theory for multi-agent systems, which has been widely applied to multi-robot formation tasks. This has led to the development of centralized leader–follower approaches, virtual structure methods, and various distributed methods. With the continuous research on consensus theory, various orderly formation methods for multi-robot formation have been developed [8,9,10,11].

It is essential to note that the Vicsek model based on the Boids rules only reproduces the polarized phenomenon in fish schools. In addition, typical ordered collective motion in fish schools also includes phenomena such as milling and bait balls. Some studies have explored models that do not adhere to the Boids rules and have achieved various group patterns. Romanczuk et al. developed a biologically motivated model based on only pursuit and escape interactions, which achieved spatial migration and vortex-like structures [12]. Similarly, Strömbom et al. proposed a model based solely on mutual attraction, which formed structures resembling milling and chain rotations by limiting the individual’s field of view [13]. Barberis proposed a group model based on position attraction, introducing a conical field of view, which resulted in linear structures, rod-like structures, milling-like structures, and gas-like structures by changing the opening angle of the cone [14]. Robert et al. presented a model with short-range velocity matching and long-range anti-alignment rules. This model generated directed ordered states, periodic vortex patterns, and medium-scale turbulence, resembling observations of swimming bacteria in dense suspensions [15]. Bastein et al. proposed a general collective model based on visual projection, which accounted for visual occlusion without requiring explicit velocity matching. By varying model parameters, this model exhibited behaviors such as aggregation, polarization, milling, and swarming under different conditions [16]. In our previous research, we proposed a fellow-following principle, established a collective model, and quantitatively compared it with the real fish school, demonstrating the similarity between the model and the real fish school [17].

**Figure 1:**
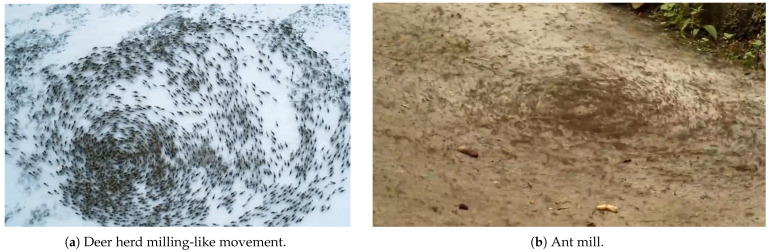
Photographs of animal milling-like movements.

However, most studies mentioned above have yet to extend the models into multi-robot formation applications. This paper aims to apply the models established in previous works to multi-robot formation, not only to achieve bio-inspired multi-robot formation but also to delve deeper into the mechanism of fish school’s milling behavior. In fact, as depicted in Figure 1, milling, or the so-called circular group motion, is not exclusive to fish. It is prevalent among various animal groups, including ants [18], chickens, ducks [19], cows, and sheep, among other animals [20]. Therefore, further investigation of the general mechanism behind such milling-like motion holds significance both in enhancing our understanding of animal collective behavior and inspiring robotic swarm formation. On the one hand, in terms of animal behavior, while the previously proposed model accomplishes the group milling, it does not explicitly elaborate on its formation mechanism or explain why milling structures take on a circular shape [17]. This is due to the self-organizing nature of the model’s process, resulting in a certain level of randomness in the outcomes. Establishing a direct causal relationship between the proposed rules and the milling motion is challenging. Thus, in pursuit of a deeper understanding of animal collective mechanisms, proposing a group model with a concise mathematical representation becomes necessary [21]. On the other hand, within the field of biomimetics, entirely self-organized group models can not be directly applicable to robot formation tasks, especially when dealing with a small robot group.

Considering these two points, the proposed model was analyzed and abstracted, leading to the development of the multi-robot circular formation method presented in this paper. The main contributions of this paper can be summarized as follows:

1. Inspired by fish milling and our previous model, it is assumed that the formation of milling is the cause of the circular communication topology and forward-following rule. Based on the proposed hypothesis, the first-order and second-order kinematics models are established, and the convergence characteristics are analyzed.
2. Based on the second-order kinematics model, combined with the robotic fish dynamics model, a robotic fish circular formation controller is proposed, and the simulation and multi-robotic fish circular formation experiment are carried out.

The remainder of this paper is organized as follows. The kinematics model and its analysis are presented in Section 2. In Section 3, the circular formation method based on the dynamics model of robotic fish is established and verified by simulation. Several groups of circular formation experiments were carried out based on robotic fish in Section 4, followed by a discussion in Section 5.

## 2. Kinematics Models

Inspired by the phenomenon of fish school and the group model proposed in our previous research, this section presents a group control protocol designed for kinematics models in a circular communication topology context. In the communication topology, where agents form a circular chain, each agent communicates with its neighboring agents in the front (or front and back) and adheres to the following rule. Through simulation, this protocol achieves a circular formation in which all agents are evenly distributed along the circumference. Protocol parameters determine the convergence radius of the circle, and the center position is related to the system’s initial state. Agents only communicate with their adjacent neighbors, rendering the proposed control protocol highly scalable. It can form a circular formation with as few as three agents to an infinite number of agents. The formation approach holds significant inspirational value for circular formation tasks involving robots. Interestingly, upon further literature review, we discovered a rule-based circular formation approach that, while slightly different in its mathematical formulation compared to our approach, shares a similar underlying concept. These studies will be mentioned in the subsequent stability analysis.

### 2.1. First-Order Kinematics Model

**Figure 2:**
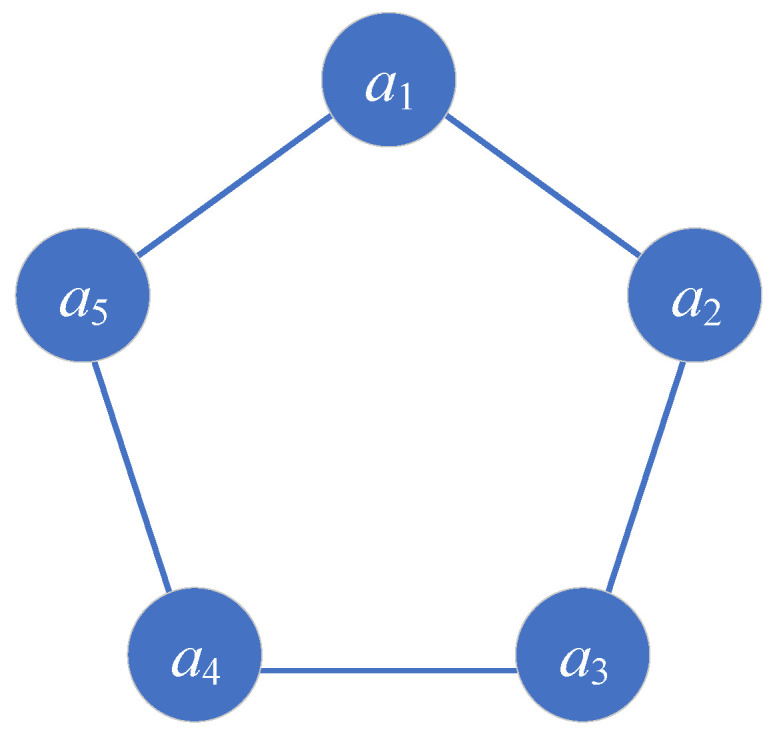
Communication topology of the first-order model.

Considering the scenario in which agents follow a first-order kinematics model, define $N_{all}$ as a set of *n* agents. This group of agents exhibits a circular communication topology. In a system composed of five agents, as shown in Figure 2, the agents are sequentially connected, forming a closed loop and creating a circular undirected graph. Information propagates between adjacent agents. The communication topology of the agents is represented by a connectivity matrix *A*:

(1) $$A=\left[\begin{array}{ccccc} 0 & 1 & 0 & 0 & 1\\ 1 & 0 & 1 & 0 & 0\\ 0 & 1 & 0 & 1 & 0\\ 0 & 0 & 1 & 0 & 1\\ 1 & 0 & 0 & 1 & 0 \end{array}\right].\;$$

In the kinematics model, the agents are treated as particles without collisions and orientations. Their state is represented by x, and, in this paper, the agents move within a two-dimensional plane, with the state x being a point in that plane. The state update equation for the agents is depicted in Formula (2).

(2) $$\begin{array}{c} \left\{\begin{array}{c} x_{t+1}^{i}=\;x_{t}^{i}+u_{t}^{i}\\ \left\{\begin{array}{c} u_{t}^{i}=\zeta \left(x_{t}^{i-1},x_{t}^{i},x_{t}^{i+1}\right),i\in \left[2,3,\ldots ,n-1\right],\\ u_{t}^{i}=\zeta \left(x_{t}^{n},x_{t}^{i},x_{t}^{i+1}\right),i=1,\\ u_{t}^{i}=\zeta \left(x_{t}^{i-1},x_{t}^{i},x_{t}^{1}\right),i=n. \end{array}\right. \end{array}\right. \end{array}$$

where $x_{t}^{i}$ represents the position of agent *i* at time *t*, $u_{t}^{i}$ indicates the control input for agent *i* at time *t*, and ζ is the control protocol function that depends on the positions of agents i−1, *i*, and i+1 at time *t*. For the first-order model, the control protocol $\zeta (x_{t}^{i-1},x_{t}^{i},x_{t}^{i+1})$ for the *i*-th agent is defined as follows:

(3) $$\begin{array}{c} \left\{\begin{array}{c} \zeta \left(x_{t}^{i-1},x_{t}^{i},x_{t}^{i+1}\right)=v_{c}\cdot \delta^{i*},\\ \delta^{i}=k_{f}\cdot \delta_{f}^{i}+\left(1-k_{f}\right)\cdot \delta_{b}^{i},\\ \delta_{f}^{i}=x_{t}^{i+1}-x_{t}^{i},\\ \delta_{b}^{i}=x_{t}^{i}-x_{t}^{i-1}. \end{array}\right. \end{array}$$

where $\delta_{f}^{i}$ is defined as the forward-following vector of agent *i*, $\delta_{b}^{i}$ represents the backward-repulsion vector of agent *i*, and $\delta^{i}$ is the weighted sum of these two vectors. $k_{f}$ indicates the weight for the forward-following vector, and $\delta^{i*}$ is the normalized unit direction vector of $\delta^{i}$. The motion step size for each update of the agent is a constant $v_{c}$.

### 2.2. Second-Order Kinematics Model

**Figure 3:**
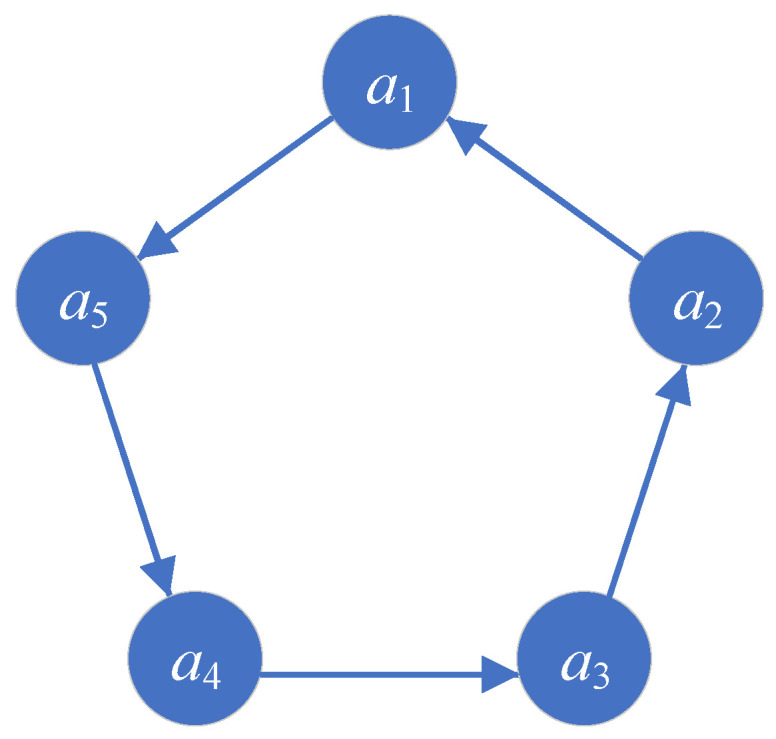
Communication topology of the second-order model.

Considering the case in which agents follow a second-order motion model, the set of agents $N_{all}$ exhibits a circular communication topology. In a system composed of five agents, as illustrated in Figure 3, the agents are connected sequentially, forming a closed loop and creating a circular directed graph. Information is transmitted from the forward agents to the adjacent agents behind them. The communication topology of the agent set can be represented by a connectivity matrix *A*:

(4) $$A=\left[\begin{array}{ccccc} 0 & 1 & 0 & 0 & 0\\ 0 & 0 & 1 & 0 & 0\\ 0 & 0 & 0 & 1 & 0\\ 0 & 0 & 0 & 0 & 1\\ 1 & 0 & 0 & 0 & 0 \end{array}\right].\;$$

In terms of the second-order case, consider a second-order unicycle model for the agents’ motion. The agents move within a two-dimensional plane, and their state is represented by x and v, where x is the position and v is the velocity. The state update equation for the agents is as follows:

(5) $$\begin{array}{c} \left\{\begin{array}{c} x_{t+1}^{i}=\;x_{t}^{i}+v_{c}\cdot v_{t+1}^{i*},\\ v_{t+1}^{i}=u_{t}^{i},\\ \left\{\begin{array}{c} u_{t}^{i}=\zeta \left(x_{t}^{i},x_{t}^{i+1},v_{t}^{i}\right),i\in \left[1,2,\ldots ,n-1\right],\\ u_{t}^{i}=\zeta \left(x_{t}^{i},x_{t}^{1},v_{t}^{i}\right),i=n. \end{array}\right. \end{array}\right. \end{array}$$

where $x_{t}^{i}$ and $v_{t}^{i}$ are the position and velocity direction of agent *i* at time *t*. $v^{i*}$ represents the unit vector of $v^{i}$. $u_{t}^{i}$ denotes the control input for agent *i* at time *t*. $v_{c}$ indicates the motion step size for the agent. ζ is the control protocol for the second-order motion model, which depends on the position and velocity of agent *i* at time *t* and the position of agent *i*+1.

For the second-order model, the control protocol ζ for the *i*-th agent is defined as:

(6) $$\begin{array}{c} \left\{\begin{array}{c} \delta^{i}=k_{f}\cdot \delta_{f}^{i*}+\left(1-k_{f}\right)\cdot v_{t}^{i*}\\ \delta_{f}^{i}=x_{t}^{i+1}-x_{t}^{i} \end{array}\right. \end{array}$$

where $\delta_{f}^{i}$ represents the forward-following vector of agent *i*. $\delta^{i}$ indicates the weighted sum of $\delta_{f}^{i}$ and the normalized direction vector $v^{i*}$. $k_{f}$ is the weight for the forward-following vector. $\delta^{i*}$ represents the normalized direction vector of $\delta^{i}$.

### 2.3. Stability Analysis of Kinematics Models

The presented first-order and second-order models both adhere to a circular communication topology and the forward-following rule. In the context of the second-order model, it is evident from Equation (7) that, at each step, it weights the heading vector of the forward agent and its own to derive the subsequent updating direction, which can be expressed as follows:

(7) $$\begin{array}{c} \begin{array}{c} \phi_{t+1}^{i}\in \left(\min\left(\phi_{t}^{i},\phi_{t}^{i+1}\right),\max\left(\phi_{t}^{i},\phi_{t}^{i+1}\right)\right) \end{array} \end{array}$$

where $\phi_{t+1}^{i}$ represents the direction angle of the *i*-th agent at time *t*+1, $\phi_{t}^{i}$ and $\phi_{t}^{i+1}$ denotes the direction angle of the *i*-th and *i*+1-th agent at time *t*, respectively.

The first-order model can be seen as a specific case of the second-order model, where the difference between the positions of the *i*-th agent and *i*−1-th agent is defined as the direction for agent *i*. As a result, the first-order and second-order models are unified, consistent with the concept of nonlinear pursuit equations proposed in the related works [22,23,24]. Therefore, the stability of the introduced first-order and second-order models can be justified using the stability analysis provided in their paper. Referring to Lemma 4 in Marshall’s paper, it can be inferred that agents asymptotically converge to a regular n-sided polygon, implying that both the first-order and second-order kinematics models proposed here achieve uniform convergence on the circular trajectory [22].

### 2.4. Convergent Circle Analysis for First-Order Model

**Figure 4:**
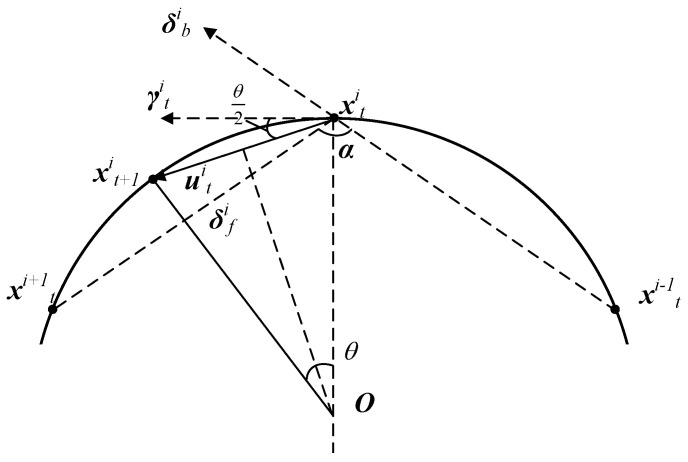
Schematic diagram of the convergent circle of the first-order model.

Simulation experiments and stability analysis indicate that for the first-order kinematics model, when the forward-following weight $k_{f}$ is less than or equal to 0.5, the model fails to converge. In fact, as can be seen from Figure 4, even if the system eventually converges to a circle, when $k_{f}$ is less than or equal to 0.5, the position of the *i*-th agent at the next moment will definitely fall outside the convergent circle instead of on the circle and will eventually be far away from the convergent circle. While $k_{f}$ is more significant than 0.5, the agents converge to a circle with a fixed center, forming a regular *n*-sided polygon evenly distributed along the convergent circle [22]. When the model’s step size $v_{c}$ and the forward-following weight $k_{f}$ are determined, the radius of the convergent circle is also determined. Based on the geometric relationship between agents during convergence, the following derivation is conducted.

Based on the stability analysis, the agents converge to a circle with a fixed center, denoted as circle *O*, and the agents are positioned to form a regular *n*-sided polygon on this circle. The schematic diagram of the convergent circle is shown in Figure 4. In the diagram, the solid arc represents the convergent circle with center O. $x_{t}^{i}$, $x_{t}^{i-1}$, and $x_{t}^{i+1}$ are located on the convergent circle, representing the positions of agent *i*, its neighbor *i*−1, and its neighbor *i*+1 at time *t*, respectively. Dashed lines represent the connections between them. $x_{t+1}^{i}$ represents the position of agent *i* at time *t*+1, and the connection between $x_{t}^{i}$ and $x_{t+1}^{i}$ is represented by a solid line. The angle $∠\overset{⌢}{x_{t}^{i}Ox_{t+1}^{i}}$ corresponds to the angle that the agent rotates along the convergent circle in one time step, denoted as θ. The distance covered by the agent in each time step is constant and denoted as $v_{c}$, so $\bar{x_{t}^{i}x_{t+1}^{i}}=v_{c}$.

Indeed, due to the movement of agents along circle *O*, $\bar{x_{t}^{i}x_{t+1}^{i}}$ is a chord on the circle, and θ represents the corresponding angle along the circumference. Based on this, the radius of the convergent circle can be determined as

(8) $$\begin{array}{c} r=\frac{v_{c}}{2\sin(\frac{\theta }{2})}. \end{array}$$

Defining the tangent vector of point $x_{t}^{i}$ on the convergent circle as $\gamma_{t}^{i}$, its direction aligns with $\delta_{f}^{i}+\delta_{b}^{i}$. Based on the geometric relationship between $u_{t}^{i}$ and the vector $\gamma_{t}^{i}$, the angle between them is θ/2, which can be calculated using the following formula:

(9) $$\begin{array}{c} \frac{\theta }{2}=\arccos\left(\frac{u_{t}^{i}\cdot \gamma_{t}^{i}}{\left|u_{t}^{i}\right|\left|\gamma_{t}^{i}\right|}\right). \end{array}$$

where $u_{t}^{i}=k_{f}\delta_{f}^{i}+\left(1\;-\;k_{f}\right)\delta_{b}^{i}$, $\gamma_{t}^{i}=\delta_{f}^{i}+\delta_{b}^{i}$. Then, expanding this formula, we have

(10) $$\begin{array}{c} \frac{\theta }{2}\;=\;\arccos\left(\frac{k_{f}{\left|\delta_{f}^{i}\right|}^{2}\;+\;\left(1\;-\;k_{f}\right){\left|\delta_{b}^{i}\right|}^{2}\;+\;\cos\left(\pi \;-\;\alpha \right)\left|\delta_{f}^{i}\right|\left|\delta_{b}^{i}\right|}{\sqrt{\left({k_{f}}^{2}{\left|\delta_{f}^{i}\right|}^{2}\;+\;{\left(1-k_{f}\right)}^{2}{\left|\delta_{b}^{i}\right|}^{2}\;+\;2k_{f}\left(1\;-\;k_{f}\right)\cos\left(\pi \;-\;\alpha \right)\left|\delta_{f}^{i}\right|\left|\delta_{b}^{i}\right|\right)\left({\left|\delta_{f}^{i}\right|}^{2}\;+\;{\left|\delta_{b}^{i}\right|}^{2}\;+\;2\cos\left(\pi \;-\;\alpha \right)\left|\delta_{f}^{i}\right|\left|\delta_{b}^{i}\right|\right)}}\right) \end{array}$$

where α is the angle $∠\overset{⌢}{x_{t}^{i+1}x_{t}^{i}x_{t+1}^{i-1}}$ formed by $\delta_{f}^{i}$ and $\delta_{b}^{i}$, and, by using the polygon interior angle sum formula, we obtain

(11) $$\begin{array}{c} \alpha =\frac{(n-2)\pi }{n} \end{array}$$

Substituting Formula (11) into Formula (10), it can further simplified by using basic properties of trigonometric functions as

(12) $$\begin{array}{c} \theta =2\arccos(\frac{1+\cos(\pi \;-\;\alpha )}{\sqrt{\left(2+2\cos\left(\pi \;-\;\alpha \right)\right)\left({k_{f}}^{2}+{\left(1-k_{f}\right)}^{2}+2k_{f}\left(1-k_{f}\right)\cos\left(\pi \;-\;\alpha \right)\right)}}). \end{array}$$

Hence, the radius of the convergent circle and the convergent adjacent distance are calculated by the following formulas:

(13) $$\begin{array}{c} \left\{\begin{array}{cc} & r_{\infty }=\frac{v_{c}}{2\sin(\frac{\theta }{2})}\\ & \Delta d_{\infty }=2r_{\infty }\sin\left(\frac{\pi }{n}\right) \end{array}\right.. \end{array}$$

**Figure 5:**
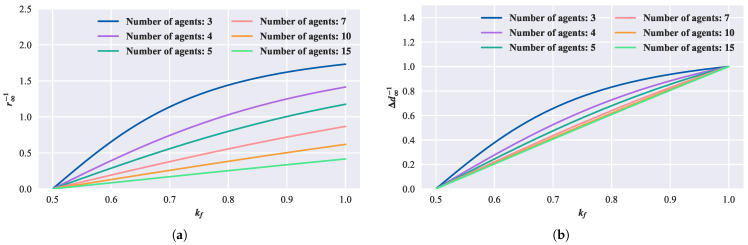
Relationship between convergent circle and parameters in the first-order model. (**a**) Relationship between convergent circle radius, $k_{f}$, and the number of agents. (**b**) Relationship between convergent adjacent distance, $k_{f}$, and the number of agents.

By choosing a step size of $v_{c}=$1, from Formula (13), the relationship between $k_{f}$ and the parameters of the convergent circle is derived, as shown in Figure 5. In this study, the distance between neighboring agents during convergence is defined as the convergence adjacent distance. The radius and the convergence adjacent distance can be used to characterize the properties of the convergent circle. For the convenience of indication, the vertical axis in Figure 5a represents the reciprocal of the radius of convergent circle $r_{\infty }$. Figure 5b represents the reciprocal of the convergent adjacent distance $\Delta d_{\infty }$. It can be observed that, with a constant number of agents, as the forward-following weight $k_{f}$ increases, the radius of the convergent circle monotonically decreases, and the convergent adjacent distance also decreases. When $k_{f}=$1, the convergent adjacent distance is independent of the number of agents and remains at one. Under the same $k_{f}$, with an increasing number of agents, the convergent circle becomes larger, and the convergent adjacent distance also increases but approaches a limit.

### 2.5. Convergent Circle Analysis for Second-Order Model

**Figure 6:**
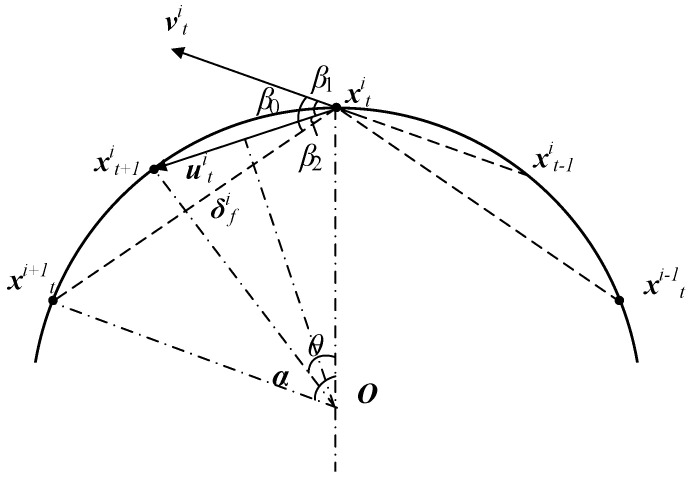
Schematic diagram of convergent circle of second-order model.

For the second-order case, simulations demonstrate that for the second-order kinematics model given in Equations (5) and (7), the model can converge when the forward-following weight $k_{f}\in \left(0,1\right]$. Similar to the first-order model, the agents converge to a fixed-center circle, and the agents form a regular *n*-sided polygon evenly distributed on the converging circle. When the model’s step length $v_{c}$ and the forward-following weight $k_{f}$ are determined, the final converging circle’s radius is also determined. Similar to the derivation for the first-order model, based on the schematic diagram of the converging circles of neighboring agents at two consecutive time steps during the convergence of the second-order model, as shown in Figure 6.

As shown in Figure 6, the intelligent agents converge to the circle O, forming a regular *n*-sided polygon. Points $x_{t-1}^{i}$, $x_{t}^{i}$, $x_{t+1}^{i}$, $x_{t}^{i-1}$, and $x_{t}^{i+1}$ represent the positions of agent *i* and its neighbors at times t−1, *t*, and t+1, respectively, which are located on the convergent circle. Thus, $v_{t}^{i}$ is collinear with $\bar{x_{t-1}^{i}x_{t}^{i}}$. $∠\overset{⌢}{x_{t}^{i}Ox_{t+1}^{i}}$ represents the angle that an agent rotates on the convergent circle within a one-time step, denoted as θ. Since the distance the agents move in each time step is constant, equal to $v_{c}$, it follows that $\bar{x_{t}^{i}x_{t+1}^{i}}=v_{c}$. In addition, due to the agents’ movement on the circle O, the distance $\bar{x_{t}^{i}x_{t+1}^{i}}$ corresponds to a chord, and θ corresponds to the central angle subtended by this chord. Hence, the formula for the convergent circle’s radius matches the one in Equation (8). Denoting the central angle corresponding to the chord $\bar{x_{t}^{i}x_{t}^{i+1}}$ as α, we have

(14) $$\begin{array}{c} \alpha =\frac{2\pi }{n}. \end{array}$$

Then, denote the angle between the velocity direction vector $v_{t}^{i}$ of agent *i* at time *t* and the forward-following vector $\delta_{f}^{i}$ of agent *i* at time *t* as $\beta_{0}$. Using the second-order control protocol from Equations (5) and (7), we can derive

(15) $$\begin{array}{c} \beta_{0}=\arccos\left(\frac{v_{t}^{i}\cdot u_{t}^{i}}{\left|v_{t}^{i}\right|\left|u_{t}^{i}\right|}\right), \end{array}$$

(16) $$\begin{array}{c} u_{t}^{i}=k_{f}\cdot \delta_{f}^{i}+\left(1-k_{f}\right)\cdot v_{t}^{i} \end{array}$$

Further, we have

(17) $$\begin{array}{c} \beta_{1}=\arccos\left(\frac{k_{f}v_{t}^{i}\cdot \delta_{f}^{i}+\left(1-k_{f}\right){\left|v_{t}^{i}\right|}^{2}}{\sqrt{k_{f}^{2}{\left|\delta_{f}^{i}\right|}^{2}+{\left(1-k_{f}\right)}^{2}{\left|v_{f}^{i}\right|}^{2}+2k_{f}\left(1-k_{f}\right)v_{t}^{i}\cdot \delta_{f}^{i}}}\right) \end{array}$$

where $\beta_{0}$ is the angle between $v_{t}^{i}$ and $\delta_{f}^{i}$, and $\beta_{2}$ is the angle between $u_{t}^{i}$ and $\delta_{f}^{i}$. Clearly, $\beta_{0}=\beta_{1}+\beta_{2}$. Using the property of the sum of interior angles in a triangle, we obtain

(18) $$\begin{array}{c} \beta_{2}=\frac{\alpha -\theta }{2}. \end{array}$$

Furthermore, at time t+1, the velocity direction of agent *i* has shifted to vector $u_{t}^{i}$; therefore, θ=β. In addition, considering that $v_{t}^{i}$ and $\delta_{f}^{i}$ are unit vectors, Equation (17) can be simplified as

(19) $$\begin{array}{c} \theta =\arccos(\frac{k_{f}\cos\left(\frac{\alpha +\theta }{2}\right)+\left(1-k_{f}\right)}{\sqrt{k_{f}^{2}+{\left(1-k_{f}\right)}^{2}+2k_{f}\left(1-k_{f}\right)\cos\left(\frac{\alpha +\theta }{2}\right)}}). \end{array}$$

Equation (19) is an implicit equation that can be solved numerically to obtain the angle θ by using a solver. Then, the calculation of the radius of the convergent circle and the convergence adjacent distance follows the same approach as in Equation (13).

**Figure 7:**
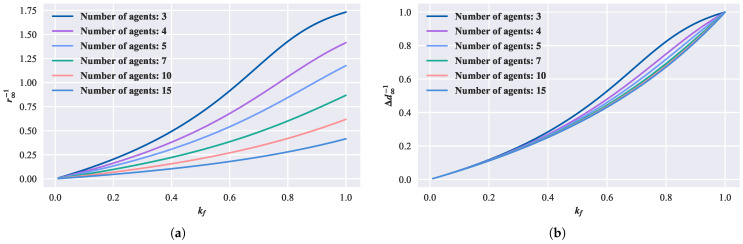
Relationship between convergent circle and parameters in the second-order model. (**a**) Relationship between the radius of convergent circle, $k_{f}$, and the number of agents. (**b**) Relationship between convergence adjacent distance, $k_{f}$, and the number of agents.

By selecting a step length $v_{c}=1$, the relationship between $k_{f}$ and the convergent circle’s parameters can be obtained, as shown in Figure 7. In Figure 7a, the relationship between $k_{f}$ and the reciprocal of the convergent circle radius is depicted, while Figure 7b shows the relationship between $k_{f}$ and the reciprocal of the adjacent distance during convergence. As can be observed, similar to the first-order model, the convergent circle size decreases with an increasing forward-following weight $k_{f}$. Furthermore, under the same $k_{f}$, more agents result in a larger convergent circle.

### 2.6. Convergent Speed Analysis of the First-Order Model

Convergence speed is a significant metric in multi-agent formation tasks, particularly in multi-robot formation. Setting control parameters reasonably and dynamically is essential to ensure the model converges quickly to the target circle. To quantify the relationship between convergence speed and model parameters, this section comprehensively analyzes convergence speed under various parameters.

**Figure 8:**
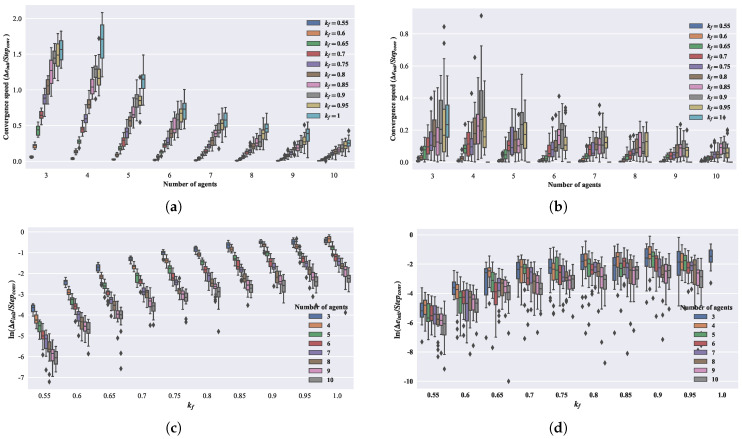
Relationship between the number of agents and $k_{f}$ with convergence speed for first-order model. (**a**) Convergence speed of the adjacent distance. (**b**) Convergence speed of the polygon angle. (**c**) Natural logarithm of the convergence speed of the adjacent distance. (**d**) Natural logarithm of the convergence speed of the polygon angle.

Simulations were conducted by varying parameters and the number of agents. The number of agents *n* and parameter $k_{f}$ were selected from the Cartesian product of the following two sets: n∈[3,4,5,6,7,8,9,10] and $k_{f}\in \left[0.55,0.60,0.65,0.70,0.80,0.90,1\right]$. Each set of parameters was repeated 20 times. The convergence speed was calculated using the following formula:

(20) $$\begin{array}{c} e_{\epsilon }=\frac{e_{0}}{Step_{\epsilon }} \end{array}$$

where ϵ denotes the convergence threshold, in this section, ϵ=0.001. $e_{0}$ represents the initial average absolute error of the group, and $Step_{\epsilon }$ denotes the convergence step. From Figure 8a,b, it can be observed that with an increase in the number of agents, the overall trend of convergence speed is decreasing, while with the increase in $k_{f}$, the overall trend of convergence speed is increasing. In Figure 8c,d, taking the natural logarithm of the convergence speed shows that as the number of agents increases, the convergence speed decreases exponentially.

### 2.7. Convergent Speed Analysis for the Second-Order Model

**Figure 9:**
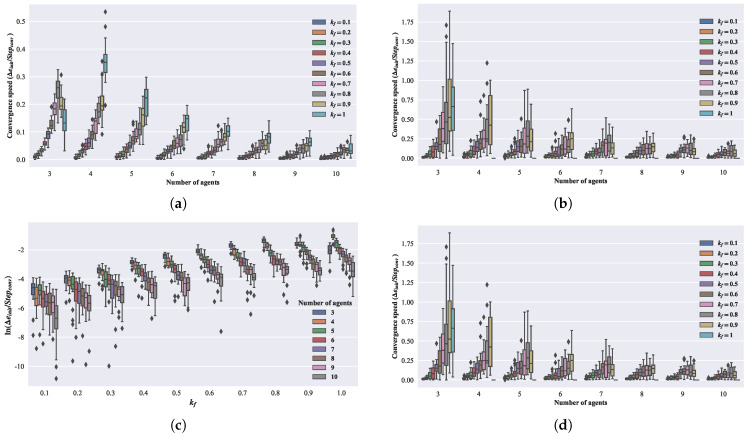
Relationship between the number of agents and $k_{f}$ with convergence speed for second-order model. (**a**) Convergence speed of the adjacent distance. (**b**) Convergence speed of the polygon angle. (**c**) Natural logarithm of the convergence speed of the adjacent distance. (**d**) Natural logarithm of the convergence speed of the polygon angle.

In the subsequent part, we will perform a convergence speed analysis for the second-order model. Similar to the analysis of convergence speed for the first-order model, the model’s convergence speed is calculated based on Equation (20), with ϵ set to 0.001. The number of agents *n* and the parameter $k_{f}$ are chosen from the Cartesian product of the following two sets: n∈[3,4,5,6,7,8,9,10] and $k_{f}\in \left[0.55,0.60,0.65,0.70,0.80,0.90,1\right]$. Each set of parameters is repeated in 20 simulations. In Figure 9a,b, it can be observed that with an increase in the number of agents, the overall trend in the convergence speed is decreasing, while, with an increase in $k_{f}$, the overall trend in the convergence speed is increasing. In Figure 9c,d, taking the natural logarithm of the convergence speed shows a consistent pattern with the first-order model. As the number of agents increases, the convergence speed decreases exponentially. For the three-agents case, the convergence speed does not strictly increase with $k_{f}$ but instead shows a slight decrease when $k_{f}>0.9$.

## 3. Circular Formation Based on Dynamics Model of Robotic Fish

Although the proposed first-order model and the second-order model can both achieve the circular formation of agents, their implementations are based on the kinematics models of agents and cannot be directly applied to the formation of robots, especially for robotic fish with nonlinear dynamics. For the implementation of robotic fish formation, the dynamics characteristics need to be considered. Therefore, based on the dual-joint robotic fish dynamics model established in [25], and combined with the second-order model control protocol, a formation control scheme in a circular topology is designed.

It should be noted that the proposed method belongs to an indirect approach to circular formation, which does not directly specify the size of the target circle but determines it through model parameters. The size of the convergent circle is related to the forward-following weight $k_{f}$, the number of agents, and the step $v_{c}$. When the number of agents is fixed, $k_{f}$ determines the angle θ (corresponding to angular velocity) that agents rotate on the circle during each update, and $v_{c}$ determines the distance (corresponding to linear velocity) that agents move during each update. For the kinematics model, given the number of agents, the desired convergent circle radius, and any three out of $k_{f}$, $v_{c}$, and θ, the remaining parameter can be calculated using Equations (13) and (19). However, for the dynamics model, its linear velocity and angular velocity are constrained and cannot directly correspond to the parameters of the kinematics model. On the other hand, the dynamics model updates in units of time, with a time step of 0.01 s. The kinematics model updates in terms of a distance step, and there is no physical correspondence between “distance” and “time”. Therefore, in order to make the circular formation based on the robotic fish dynamics model match the parameters of the kinematics model when the number of agents and $k_{f}$ are the same, it is necessary to design a circular formation controller for robotic fish based on the proposed second-order model.

### 3.1. Design of Circular Formation Controller

In the defined circular formation, the angular velocity of agents during convergence in the robotic fish dynamics model is denoted as $\omega_{rob}=\eta_{ts}\theta$, and the linear velocity is denoted as $v_{rob}=\eta_{ts}v_{c}$. Here, $\eta_{ts}$ is defined as the time scaling factor, which signifies how many steps of the kinematics model correspond to one second of the dynamics model. Therefore, for the circular formation of robotic fish based on the dynamics model, controlling the swimming speed $v_{rob}$ and angular velocity $\omega_{rob}$ of the robotic fish allows us to match the parameters of the kinematics model’s convergent circle.

**Figure 10:**
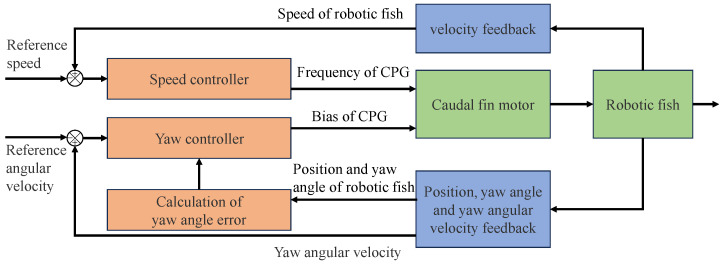
Control diagram of the robotic fish circular formation.

Due to the negligible roll and pitch movements of robotic fish when swimming in a plane, this paper focuses solely on motion control within a two-dimensional plane to simplify the complexity of the formation problem. In this context, the control of the robotic fish’s planar motion is broken down into yaw control, velocity control, and angular velocity control. Figure 10 illustrates the control system diagram for planar motion.

Velocity control aims to align the swimming speed of the robotic fish with the desired speed. The fish’s swimming speed is influenced by the amplitude, frequency, and bias of caudal fin oscillations, all of which are nonlinearly coupled. To simplify the controller complexity, assume a constant caudal fin oscillation amplitude and adjust the frequency of the caudal fin oscillations for the velocity control. A proportional integral (PI) control method is employed as follows:

(21) $$\begin{array}{c} f_{t}=k_{pft}v_{e}+k_{ift}\int v_{e}\;dt \end{array}$$

where $f_{t}$ denotes the oscillation frequency of the caudal fin’s central pattern generator (CPG), and $k_{pft}$ and $k_{ift}$ are controller parameters. $v_{e}$ represents the velocity error.

The yaw control ensures that the robotic fish can track a target direction within the horizontal plane. When the desired yaw angle remains constant, the fish’s direction of motion should stabilize at the desired yaw angle. Yaw control is achieved using a proportional-derivative (PD) controller:

(22) $$\begin{array}{c} b_{t}=k_{pbt}\psi_{e}+k_{dbt}\left(\dot{\psi }-\omega_{rob}\right) \end{array}$$

where $b_{t}$ is the oscillation bias of caudal fin, and $k_{pbt}$ and $k_{dbt}$ are controller parameters. ψ represents the yaw angle, and $\psi_{e}$ denotes the yaw angular error, which corresponds to the azimuthal difference between $\delta^{i}$ and the robot’s swimming direction $v_{t}^{i*}$ in Equation (7).

For circular formation, since the desired yaw angle is continually changing, adjusting the yaw angle velocity to stabilize at $\omega_{rob}$ involves dynamically modifying $k_{dbt}$ through integral control action to eliminate angular velocity residuals:

(23) $$\begin{array}{c} k_{dbt}=k_{dbt_{-1}}-k_{c}\left(\dot{\psi }-\omega_{rob}\right)\Delta t \end{array}$$

where $k_{dbt_{-1}}$ represents $k_{dbt}$ at the previous time step, $k_{c}$ is the controller parameter, and Δt denotes the control cycle.

The velocity and yaw controller enables the robotic fish to form a convergent circle and maintain stable movement along the circular path. By automatically adjusting the parameter $k_{dbt}$ through yaw control, the angular velocity error can be eliminated, ensuring that the convergent circle’s parameters match the calculation by Equations (13) and (19).

**Figure 11:**
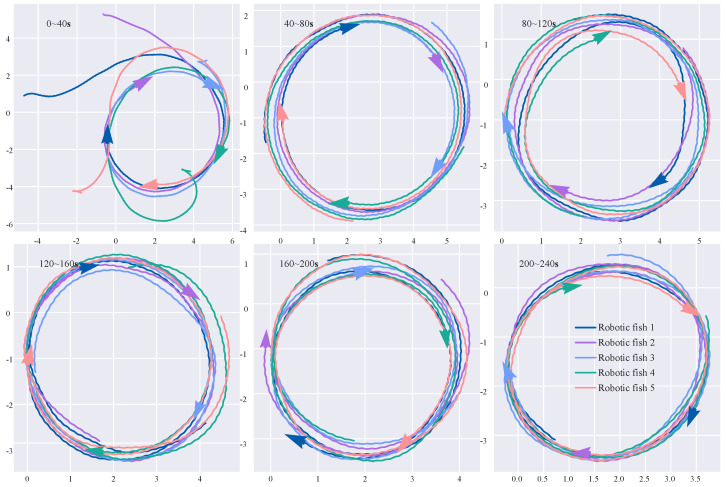
Circular formation simulation of five robotic fish.

Figure 11 shows snapshots of the paths of five robotic fish in a circular formation with parameter $k_{f}=0.3$, a kinematics model step size of 0.5, and the scaling factor $\eta_{ts}=0.6$.

#### 3.1.1. Simulation Results of Changing Speed and Scaling Factor

This part will discuss whether the convergent circle of the dynamics model matches the theoretical value. The proposed circular formation control algorithm will be validated from four aspects: the number of robotic fish, the forward-following parameter $k_{f}$, the step $v_{c}$, and the scaling factor $\eta_{ts}$.

**Figure 12:**
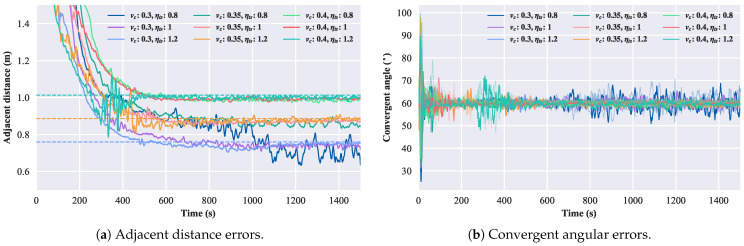
Error diagrams of changing speed and scaling factor.

Firstly, verify whether the size of the convergent circle matches the formula given in Equation (13) when the step $v_{c}$ and scaling factor $\eta_{ts}$ vary. The steps are set to [0.3,0.35,0.4] m/s, and the scaling factors $\eta_{ts}$ are in the range of [0.8,1,1.2]. $k_{f}$ is set to 0.5, and the number of robotic fish is three. The simulation results are depicted in Figure 12. The dashed lines in the figure represent the nearby convergent distance calculated according to Equation (13). The results from Figure 12 indicate that the scaling factor $\eta_{ts}$ does not affect the size of the convergent circle, while the step $v_{c}$ is directly proportional to the size of the convergent circle.

#### 3.1.2. Simulation Results of Changing $k_{f}$

**Figure 13:**
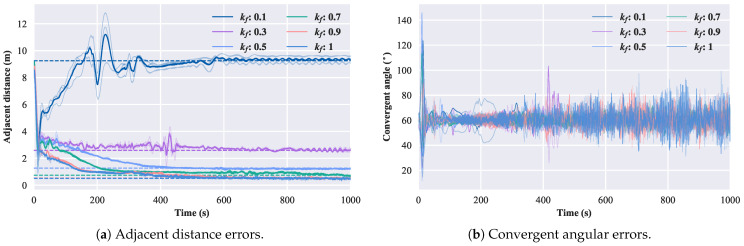
Error diagram of changing $k_{f}$.

Then, we will study the relationship between the size of the convergent circle and the theoretical value when $k_{f}$ varies. The swimming speed of the robotic fish is set to 0.5 m/s, and $k_{f}$ is varied within the range of [0.1,0.3,0.5,0.7,0.9,1], corresponding to the angular velocities $\omega \in [5.36^{\circ },19.27^{\circ },40^{\circ },71.92^{\circ },107.86^{\circ },120^{\circ }]$. Considering the thresholds of the robotic fish’s swimming speed and turning angular velocity, the scaling factor $\eta_{ts}$ is set to [1,1,0.6,0.4,0.4,0.4]. The simulation results are shown in Figure 13.

The dashed lines in the figure represent the nearby convergence distance calculated based on Equation (13). These lines demonstrate that as $k_{f}$ varies, the size of the convergent circle in the robotic fish formation still conforms to Equation (13).

### 3.2. Simulation Results of Changing Number of Agents

**Figure 14:**
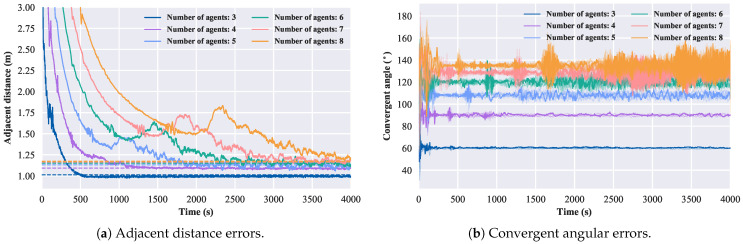
Error diagram of changing the number of agents.

Finally, we investigate the relationship between the size of the convergent circle and the theoretical values when the number of robotic fish varies. For this analysis, we set $k_{f}$ to 0.5, the step $v_{c}$ to 0.5 m/s, and vary the number of robotic fish as [3,5,7,9]. The convergence behavior is depicted in Figure 14.

In brief, by varying the number of robotic fish, the value of $k_{f}$, the step $v_{c}$, and the scaling factor $\eta_{ts}$, the results demonstrate that the proposed control approach for robotic fish formation is equivalent to the second-order kinematics model in terms of forming the convergent circle.

## 4. Circular Formation Experiments of Biomimetic Robotic Fish

**Figure 15:**
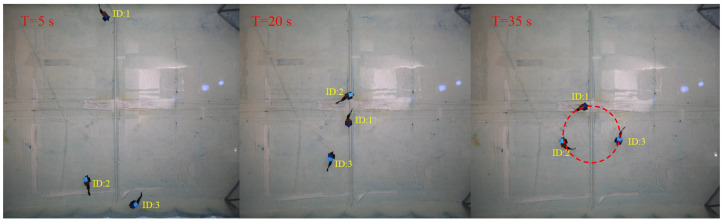
Experimental scenario of three robotic fish executing circular formation.

**Figure 16:**
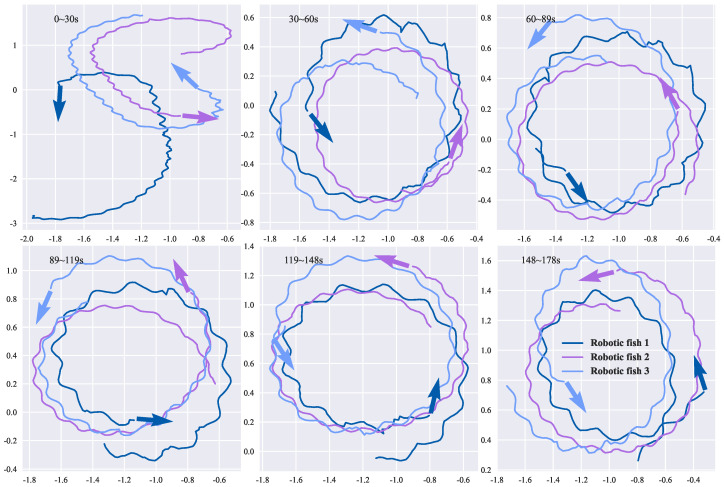
Path diagram of three robotic fish executing circular formation.

Using the two-joint biomimetic robotic fish developed in [25], a circular formation experiment was conducted with multiple robotic fish. The robotic fish takes a black koi fish as the bionic object, which is about 25 cm long, 5 cm wide, 9 cm high and weighs about 340 g. In this multi-robot circular formation experiment, the parameters were set as follows: $k_{f}=0.2$, implying that the angular velocity of the robotic fish during convergence was approximately $12^{\circ }$; the scaling factor was set to 1; the kinematics model step size was 0.12 for three robotic fish, 0.14 for four robotic fish, and 0.13 for five robotic fish, and the swimming velocity was 0.12 m/s, 0.14 m/s, and 0.13 m/s, respectively. The experimental scenario is depicted in Figure 15, and Figure 16 illustrates the paths of the three robotic fish during the experiment, with the horizontal and vertical axes measured in meters.

**Figure 17:**
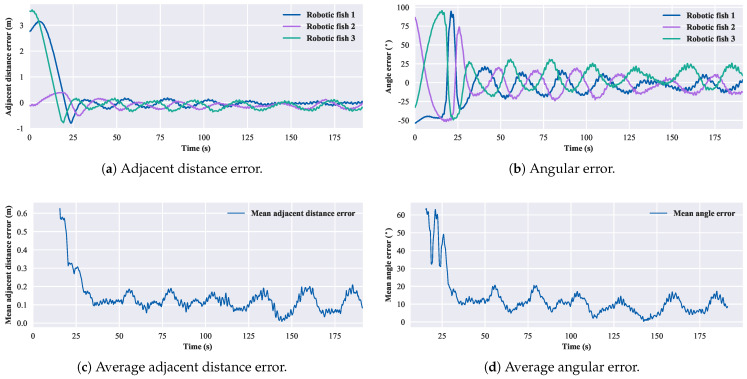
Results of the circular formation experiment of three robotic fish.

**Figure 18:**
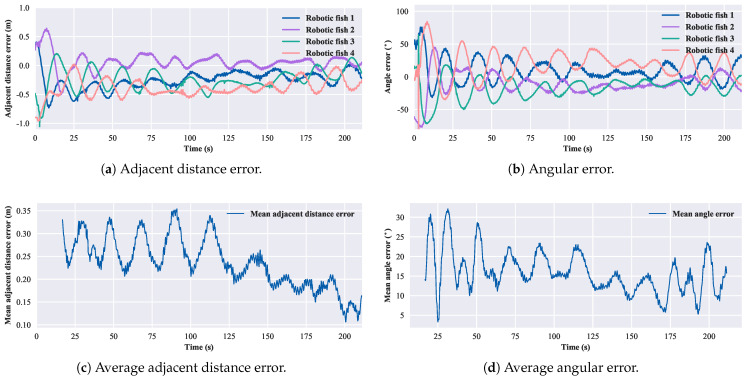
Results of the circular formation experiment of four robotic fish.

**Figure 19:**
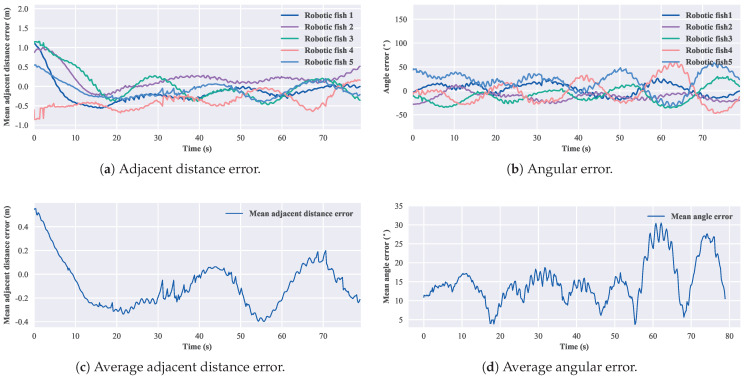
Results of the circular formation experiment of five robotic fish.

According to Equation (13), for the three-robotic-fish formation, the convergent adjacent distance is 1.02 m, and the convergent angle is $60^{\circ }$. The experimental results of the three-robotic-fish circular formation are shown in Figure 17. After 30 s, the average error in the adjacent distance is 0.12 m, and the average error in the convergent angle is $10.19^{\circ }$. For the four-robotic-fish case, the convergent adjacent distance is 1.19 m, and the convergent angle is $90^{\circ }$. The experimental results of the four-robotic-fish circular formation are shown in Figure 18. After 30 s, the average error in the adjacent distance is 0.23 m, and the average error in the convergent angle is $15.13^{\circ }$. For the five-robotic-fish formation, the convergent adjacent distance is 1.11 m, and the convergent angle is $108^{\circ }$. The experimental results of the multi-robot circular formation are shown in Figure 19. After 30 s, the average error in the adjacent distance is 0.38 m, and the average error in the convergent angle is $22.69^{\circ }$. The results verify the effectiveness of the circular formation approach.

## 5. Discussion

This study is inspired by the milling motion observed in fish schools and similar behaviors in animals. For the task of circular formation in multi-robot fish systems, we proposed a circular formation algorithm based on a circular communication topology and the following rules. Specifically, we introduced first-order and second-order circular topology control protocols based on kinematics models, enabling multiple agents to converge uniformly to a target circle. Furthermore, we designed controllers for the robotic fish based on their kinematics models, enabling the simulation of circular formation with multiple robotic fish. The fish swarm demonstrated convergence to the target circle across various parameter settings, consistently maintaining the same target circle size as the kinematics model. Furthermore, we conducted experiments involving formation with three, four, and five robotic fish, confirming the effectiveness of our algorithm on the robotic fish platform. Through mathematical analysis, simulations, and experiments, we not only showcased the proposed algorithm’s efficacy in addressing circular formation tasks for multi-robot systems but also suggested that the phenomenon of milling, commonly observed in the animal kingdom, is likely a result of creatures adhering to the following rule. This may lead to the spontaneous or externally induced formation of a stable circular communication topology, ultimately resulting in similar milling patterns across different species.

In Section 4, it can be observed that the errors in the convergence adjacent distance and angles are relatively large. This is partly due to the inherent under-actuation of the robotic fish and inconsistencies in the manufacturing of individual fish, which affect the control efficiency of the collective formation. Improving the consistency of the robotic fish and the control performance could lead to a smaller error in circular formation [26,27,28].

In the future, we will pursue research from two aspects. Firstly, we will delve further into the underlying mechanisms of animal collective behaviors, such as the bait-ball formation observed in fish schools. These widespread biological phenomena undoubtedly have principles that can be abstractly modeled. Based on the detailed study of the fish lateral line system [29], modeling, analyzing, and abstracting are carried out to validate conclusions drawn from the study of animal behaviors. Secondly, we will explore potential applications for circular formation tasks. Animals lack efficient communication and typically rely on passive perception rather than active communication for coordination. However, robots often construct wireless communication networks, offering higher communication efficiency. Animal milling behaviors are based on passive perception, which does not directly match the communication patterns of robots. Moreover, fish schooling behavior is a defense mechanism against predators, utilizing rapid rotations to lower the predators’ efficiency. However, robots do not need to replicate such behaviors in their task scenarios. Seeking suitable application scenarios for the algorithm proposed in this paper remains a significant challenge, which may provide more insight into pursuit–evasion problems of multi-agent systems [30].

## Data Availability

Data are contained within the article.
